# Technoeconomic evaluation of recent process improvements in production of sugar and high-value lignin co-products via two-stage Cu-catalyzed alkaline-oxidative pretreatment

**DOI:** 10.1186/s13068-022-02139-5

**Published:** 2022-05-04

**Authors:** Zhaoyang Yuan, Bryan D. Bals, Eric L. Hegg, David B. Hodge

**Affiliations:** 1 Department of Biochemistry & Molecular Biology, Michigan State University, 603 Wilson Road, East Lansing, MI 48824 USA; 2 Michigan Biotechnology Institute, 3815 Technology Boulevard, Lansing, MI 48910 USA; 3 Department of Chemical & Biological Engineering, Montana State University, Bozeman, MT 59717 USA; 4 Division of Sustainable Process Engineering, Luleå University of Technology, Luleå, Sweden

**Keywords:** Alkaline hydrogen peroxide pretreatment, Biorefinery, Enzymatic hydrolysis, Hybrid poplar, Biomass, Cellulosic biofuels, Lignin, Technoeconomic evaluation

## Abstract

**Background:**

A lignocellulose-to-biofuel biorefinery process that enables multiple product streams is recognized as a promising strategy to improve the economics of this biorefinery and to accelerate technology commercialization. We recently identified an innovative pretreatment technology that enables of the production of sugars at high yields while simultaneously generating a high-quality lignin stream that has been demonstrated as both a promising renewable polyol replacement for polyurethane applications and is highly susceptible to depolymerization into monomers. This technology comprises a two-stage pretreatment approach that includes an alkaline pre-extraction followed by a metal-catalyzed alkaline-oxidative pretreatment. Our recent work demonstrated that H_2_O_2_ and O_2_ act synergistically as co-oxidants during the alkaline-oxidative pretreatment and could significantly reduce the pretreatment chemical input while maintaining high sugar yields (~ 95% glucose and ~ 100% xylose of initial sugar composition), high lignin yields (~ 75% of initial lignin), and improvements in lignin usage.

**Results:**

This study considers the economic impact of these advances and provides strategies that could lead to additional economic improvements for future commercialization. The results of the technoeconomic analysis (TEA) demonstrated that adding O_2_ as a co-oxidant at 50 psig for the alkaline-oxidative pretreatment and reducing the raw material input reduced the minimum fuel selling price from $1.08/L to $0.85/L, assuming recoverable lignin is used as a polyol replacement. If additional lignin can be recovered and sold as more valuable monomers, the minimum fuel selling price (MFSP) can be further reduced to $0.73/L.

**Conclusions:**

The present work demonstrated that high sugar and lignin yields combined with low raw material inputs and increasing the value of lignin could greatly increase the economic viability of a poplar-based biorefinery. Continued research on integrating sugar production with lignin valorization is thus warranted to confirm this economic potential as the technology matures.

**Supplementary Information:**

The online version contains supplementary material available at 10.1186/s13068-022-02139-5.

## Introduction

Substantial research has been directed at developing technologies to convert lignocellulosic biomass into renewable biofuels and bio-based chemicals and materials, with the objective of facilitating the transition of the petroleum-based economy into a bioeconomy [1–3]. One focus has been the deconstruction of structural polysaccharides in the cell walls of plants to monomeric sugars that can be further processed through biological, catalytic, or chemical conversion. To achieve this goal, a wide range of chemical, physical, and biological biomass deconstruction/pretreatment technologies have been developed to improve the recovery of sugars by reducing the recalcitrance of the cell wall [2, 3]. While significant progress has been achieved, economic challenges remain [4], and consequently, identifying approaches to reduce the process cost and/or improve the product value are of great importance for commercializing this biorefinery concept.

One promising approach is to produce co-products along with biofuels that can both improve the overall economics of the process and improve the overall carbon/mass efficiency of the process. Additionally, diversification of the product portfolio of lignocellulose-to-biofuels processes is widely recognized as an opportunity both to improve the economics of these processes and to buffer against market fluctuations. Lignin is the major non-polysaccharide structural component of lignocellulosic biomass at approximately 18–30% of the total dry mass weight and, as such, represents a promising source of reduced carbon for fuels and chemicals. While many biorefining concepts consider the use of process-modified lignins as a relatively low-value fuel to provide process heat and power [5–8], if key functionalities can be preserved or process modifications of the lignin are minimized, lignin can serve as a source of renewable aromatics for a diverse range of co-product applications. As one example, process-modified lignins can be utilized as a renewable bio-based polyol in the production of polyurethanes [9, 10], which has been shown to exhibit improved biodegradability compared to the petroleum-based polyurethanes [11, 12]. As another example, lignin can serve as the raw material for functionalized aromatic monomers such as vanillin, vanillic acid, syringic acid, syringaldehyde, and *p*-hydroxybenzoic acid, which can be used as platform chemicals [13–15]. Therefore, it is important to develop an understanding of these integrated economic models of integrated biorefineries that can partition both polysaccharides and lignins into fuels and chemical co-products, thereby improving their use in the production of sugars and lignin at a low cost and minimum usage of reagents.

Lignin chemical structures, properties, and utility in specific applications are a strong function of both the biomass source and prior processing history of the lignin [16–18]. Notably, lignins may undergo significant loss of utility due to repolymerization reactions when subjected to dilute acid pretreatment or delignification during Kraft pulping [19–22]. Consequently, biomass pretreatment or fractionation technologies may be economically compelling if they are capable of yielding both a clean sugar stream for the production of biofuels and lignins that can feed multiple co-product streams, while additionally providing flexibility in the partitioning of lignin between co-product streams. Our prior work with two-stage alkaline pre-extraction followed by copper-catalyzed alkaline hydrogen peroxide pretreatment (Cu-AHP) demonstrated the potential of this technology for producing fermentable sugars at high yields for biofuel production while simultaneously recovering high-quality lignin as a co-product. We recently demonstrated nearly complete deconstruction of structural polysaccharides to monosaccharides while simultaneously solubilizing over 70% of the original lignin [23]. Importantly, this prior work also demonstrated that the recovered lignins are more suitable in co-product applications than other process-derived lignins (e.g., Kraft lignin). Firstly, we demonstrated that Cu-AHP lignins were suitable as an aromatic polyol in polyurethane resin applications and 30% more reactive with isocyanate than Kraft lignin (on the basis of equivalent aliphatic hydroxyl content) [23], making it an ideal aromatic polyol substitute in polyurethanes. Likewise, the lignins from this process could be depolymerized into potentially high-value aromatic monomers at high yields (> 30%), which is significantly higher than yields achievable from many other process-derived lignins [16, 24–26].

This present study analyzed the economic impact of these advances, considering multiple uses for lignin to determine the impact on the minimum fuel selling price (MFSP) using experimental data for different hybrid poplar processing scenarios published in our prior work [23]. The technoeconomic analysis (TEA) was performed using an Excel-based model derived from the National Renewable Energy Laboratory (NREL) model for catalytic upgrading of sugars to hydrocarbons [27]. Scenarios varying lignin utilization were analyzed to determine strategies to further improve MFSP that included the impact of varying the percentage of the lignin stream diverted from energy production to valorization into high-value products such as renewable polyols for polyurethane coating applications and depolymerization to aromatic monomers. Finally, a sensitivity analysis was conducted to identify parameters with the most significant impacts on economic performance.

## Materials and methods

### Modeling overview

**Fig. 1:**
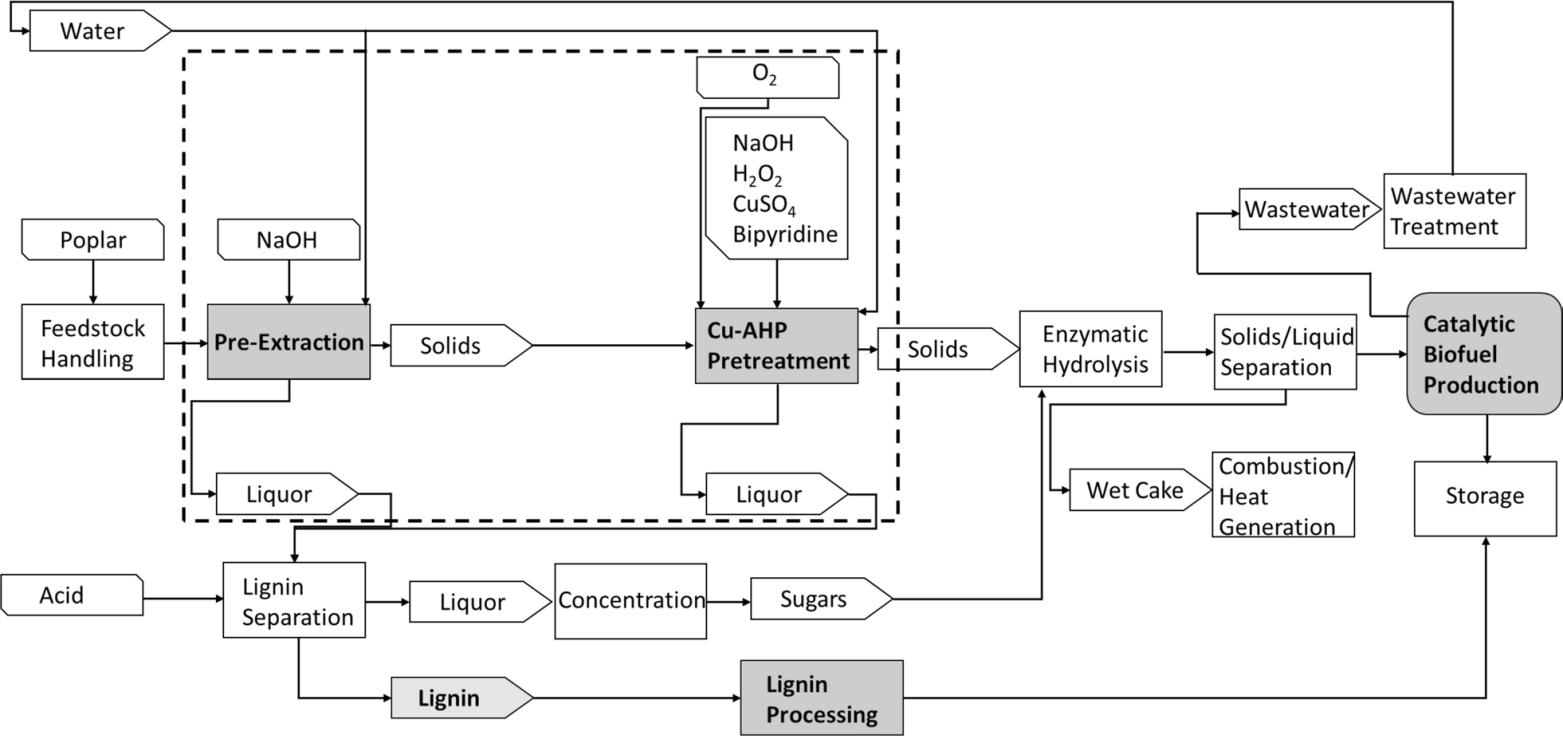
The flow diagram of the proposed two-stage alkaline pre-extraction/alkaline-oxidative pretreatment technology for poplar biorefinery

The technoeconomic model of the two-stage alkaline-oxidative pretreatment for a cellulosic biorefinery with a capacity of 2000 metric tonnes per day was developed using Microsoft Excel 2016 based on the NREL model for a process of converting corn stover to hydrocarbons [27]. In brief, our model employed hybrid poplar rather than corn stover and replaced the pretreatment module of the NREL model with the two-stage pretreatment comprising alkaline pre-extraction followed by Cu-AHP delignification described in our prior work [23]. All pieces of equipment, material streams, and major energy flows were accounted for in this model. Moreover, a material balance was used to modify the sizing of all downstream operations, and this resizing was used to determine all capital, material, and energy costs. The general scheme for mass flows for the two-stage alkaline-oxidative pretreatment with a process description and experimental process parameters is summarized in Fig. 1. The process model considered all unit operations required to transform the biomass into monomeric sugars, mainly glucose and xylose. Those operations were grouped into five major discrete units, namely feedstock preparation, alkaline pre-extraction, alkaline-oxidative pretreatment, lignin recovery and valorization, and enzymatic hydrolysis (Fig. 1). All models were based on experimental results for pretreatment process performance (biomass compositions and response to pretreatment and enzymatic hydrolysis) that were published previously [23].

### Modeling unit

#### Feedstock preparation unit

The feedstock for the modeled process was debarked hybrid poplar (*Populus nigra* var. *charkoviensis* × *caudina* cv. NE-19) with a composition of 45.5% glucan, 15.8% combined xylan, galactan, and mannan, 22.3% Klason lignin, 2.5% acid-soluble lignin, and 0.85% ash [23]. Briefly, the harvested poplar was air-dried and debarked prior to delivery. The debarked logs were subjected to size reduction comprising chipping and milling to prepare required-sized biomass for the biorefinery. The biomass loss during the size reduction process was assumed to be 1%, which was directed to the boiler for combustion for energy/heat. The cost of poplar used in the model ($55/dry tonne) includes the costs of feedstock and the delivery of processed feedstock.

#### Pretreatment unit

The two-stage pretreatment process was fully described in our earlier work [23], while pretreatment capital costs were estimated based on our previous technoeconomic model [28]. Briefly, sodium hydroxide (NaOH) and the prepared biomass feedstock were mixed in the pretreatment reactor at 10% NaOH loading on biomass (w/w based on the dry weight of the biomass) and 10% (w/v) consistency at either 90 °C or 120 °C for 1 h. The chemical composition of the alkaline pre-extracted poplar biomass was determined (Additional file 1: Table S1). For alkaline pre-extraction, the reactor was modeled as a vertical agitated vessel with a conical bottom and screw discharge and entry with a maximum size of 1000 m^3^. Following alkaline pre-extraction, the solid biomass was subjected to the second-stage alkaline-oxidative pretreatment, during which NaOH, CuSO_4_, 2,2ʹ-bipyridine (bpy), hydrogen peroxide (H_2_O_2_), and oxygen (O_2_) were added to the pre-extracted poplar for further fractionation. For the alkaline-oxidative pretreatment step, the reactor was modeled as two smaller vertical reactors in sequence, each approximately 680 m^3^, in which the O_2_ and reagents could be mixed with the pre-digested biomass. Pretreatment conditions and reagent loadings are shown in Table 1. Once all material was mixed, the slurry was digested for 12 h in vertical reactors with a maximum size of 3000 m^3^. In establishing the economic model, the biomass chips were assumed to be transported to the refinery at a cost of $55/tonne dry weight based on the lower end of the range estimated in the literature for delivered biomass costs [29]. The chips were optionally milled further to 5 mm particle size via a hammer mill at an electrical cost of 40 kWh/tonne [30]. After alkaline-oxidative pretreatment, a solid–liquid separation was performed via a pneumatic filter press with the same cost assumptions as Davis et al. scaled to the amount of solids recovered [27]. Following filtration, the liquid streams were subjected to lignin recovery through acidification to pH 2 with H_2_SO_4_ and filtration through a standard filter press.

**Table 1:** Conditions assessed for the second-stage alkaline-oxidative pretreatment

Alkaline-oxidative pretreatment parameter	Value
Temperature (°C)	80
Residence time (h)	12
CuSO_4_ dosage (wt. %)^a^	0.159
2,2'-Bipyridine (bpy) dosage (wt. %)^a^	0.156
NaOH loading (wt. %)^a^	10
H_2_O_2_ loading (wt. %)^a^	0–8
O_2_ pressure (psig)	50

^a^Based on the weight of original biomass

#### Processing liquor recovery unit

After the first alkaline pre-extraction stage, the solubilized lignin was separated and recovered through sequential acidification to pH 2 with 72% (w/w) H_2_SO_4_ and filtration through a standard filter press. In addition, we have demonstrated the dissolved carbohydrates in the alkaline pre-extraction liquor can also be directly recovered by adding the liquor into the enzymatic hydrolysis step [31]. As utilizing both the pre-extraction stream and the alkaline-oxidative pretreatment stream would result in an enzymatic hydrolysis stream that is too dilute to be viable, the remaining liquor was assumed to be concentrated via evaporation, with capital and energy costs obtained from Davis et al. and scaled to the amount of water evaporated [27]. Note that this was not performed at lab scale and thus remains to be tested. In the lab-scale work, the lignin precipitate was washed 2 times with aqueous H_2_SO_4_ (pH 2.0), and finally washed one time with cold deionized water. For the purposes of this TEA, the downstream lignin purification activities were not modeled. It was assumed that the lignin would be sold in a crude state with the price reflecting the fact that further processing may be needed. Similarly, the dissolved lignin and carbohydrates in the liquor obtained from the second alkaline-oxidative pretreatment stage were recovered in the same process. Carbohydrates solubilized from both the alkaline pre-extraction step and the second-stage alkaline-oxidative O_2_-Cu-AHP process were assumed to be utilized for enzymatic hydrolysis.

#### Enzymatic hydrolysis unit

In this process, the solid fraction, dissolved carbohydrates, water, and enzyme were mixed. An enzyme loading of 15 mg protein/g glucan (consisting of CTec3 and HTec3 at a protein ratio of 1:1) was used for this study. The enzymes were assumed to be purchased at a cost of $5/kg protein (Table 2), slightly higher than modeled in the 2011 NREL model [5]. Sugar yields were assumed to be identical to those in the laboratory experiments. The resulting sugars were modeled to be combined with purchased hydrogen (H_2_) and catalytically upgraded to hydrocarbon biofuel, while the residual enzymatic hydrolyzed solids were combusted to generate energy for the biorefinery plant (Fig. 1). All equipment downstream from the enzymatic hydrolysis were modeled from Davis et al. and scaled appropriately to the size of the streams [27].

**Table 2:** Cost and operation assumptions and parameters used in the economic model

Raw material costs	
Hybrid poplar	$55/dry metric tonne
Glycoside hydrolase enzymes	$5/kg protein
NaOH	$149/metric tonne
CuSO_4_	$1.50/kg
2,2′-Bipyridine	$30/kg
H_2_O_2_	$1.00/kg
H_2_SO_4_	$88/metric tonne
Product selling price	
Hydrocarbon biofuel	MFSP, set by solution to economic model
Lignin selling price	$0.80/kg
Biorefinery operation	
Biorefinery throughput	83.3 dry metric tonne/h
Biorefinery operation	8400 h/year
Installed capital costs	
Pre-extraction reactor (90 °C)	$2,133,000
Pre-extraction reactor (120 °C)	$2,879,000
Pretreatment reactor (no O_2_)	$15,746,000
Pretreatment reactor (with O_2_)	$28,157,000
Material handling	$4,500,000
Oxygenation	$9,590,000
Pretreatment concentration and lignin separation	$62,893,000
Enzymatic hydrolysis	$65,682,000
Catalytic conversion	$101,617,000
Wastewater treatment	$78,951,000
Storage	$5,544,000
Boiler	$39,386,000
Utilities	$4,192,000

#### Lignin recovery and valorization unit

The lignin-rich solids obtained from the processing liquors (both alkaline pre-extraction and alkaline-oxidative pretreatment liquors) were considered as a source of polyols for use in polyurethane coatings applications or used for the production of aromatic monomers. However, the system boundary of this model does not include upgrading of lignin to final products. Multiple scenarios were tested with lignin. As a base case, the solubilized lignin was assumed to be sold as-is at $0.80 per kg, approximately half the market value of polyols used for polyurethane [32]. Currently, only acid precipitation has been tested as a means of isolating the lignin from both stages of the pretreatment process, which recovered 79–80% and 31–35% of solubilized lignin during the first-stage alkaline pre-extraction and the second-stage alkaline-oxidative pretreatment, respectively. Thus, two scenarios were tested: one in which only the lignin recovered via acid precipitation is sold (with the remainder burned for energy), and one in which all solubilized lignin is assumed to be recoverable. In addition, a portion of the lignin could be depolymerized to monomers, which could be sold at a higher value than as a polyol substitute. Given the limited knowledge surrounding the value of these monomers and the cost of upgrading the lignin, we assumed a flat value of $2.00/kg for lignin to be upgraded to monomers.

### Process economic analysis

For the economic analysis a biorefinery with a throughput of 2000 dry metric tonnes of biomass per day and operated for 350 days per year and 24 h/day [5] with a summary of the key cost and operational assumptions for economic analysis presented in Table 2. Chemical raw material costs were based on estimates obtained from Alibaba.com, while process equipment units were sized based on operating conditions and the mass and energy balance from the process model. Equipment capital costs from the Davis et al. [27] model were used as a basis for estimates in the present study with appropriate scaling. In addition to the equipment purchase cost, an installation cost for each piece of process equipment was also included as installed cost. For example, the use of O_2_ as a co-oxidant required increased thickness of the reactor to tolerate high pressure. Thus, the installed cost was higher than that of the reactor used for reaction without O_2_, which was considered in the economic model (Table 2). In addition, the O_2_ was assumed to be generated from air, with cost data derived from the National Energy Technology Laboratory and scaled appropriately [33]. When O_2_ was used as a co-oxidant, the pretreatment reaction vessels were rated for 0.7 MPa to account for the O_2_ pressure and elevated temperature. Vessel thickness was adjusted based on design equations from Peters et al. [34] and the cost of the vessel adjusted accordingly. The alkaline pre-extraction vessels were assumed to be rated for 0.3 MPa if performed at 90 °C and 0.5 MPa if performed at 120 °C. Once all capital and operating costs were obtained, the minimum fuel selling price (MFSP) was obtained by fixing the 30-year net present value of the biorefinery to $0 using a 10% internal rate of return. All assumptions for calculating this rate of return are the same as in the NREL model [27].

### Sensitivity and uncertainty analysis

Given the inherent uncertainty in economic modeling, a sensitivity analysis was performed around factors identified as being either significant to the final MFSP or to the factors considered the most uncertain. The operational costs were fixed at $239.13 MM per year. Other factors were adjusted upward or downward by 25% and the model re-run to determine its impact on the MFSP. Given the high uncertainty of the value of lignin as a monomer, this value was adjusted by 50%, and only the low value is shown in order to showcase the worst-case scenario. The factors chosen and the low, medium, and high values (where applicable) are shown in Table 3.

**Table 3:** Assumptions for the sensitivity analysis

Factors	Low values	Base case	High values
Sugar usage	Sugars from pre-extraction not included	All sugars included	N/A
Lignin value as a polyol	$0.60/kg lignin	$0.80/kg lignin	$1.00/kg lignin
Named monomer price	$1.00/kg	$2.00/kg	N/A
H_2_O_2_ price	$750/tonne	$1000/tonne	$1250/tonne
Pretreatment vessel capital cost	$23.78 MM	$31.71 MM	$39.64 MM
Oxygen usage	28.1 g/kg poplar	37.5 g/kg poplar	46.9 g/kg poplar

## Results and discussion

### Overview of biomass conversion pathway

**Fig. 2:**
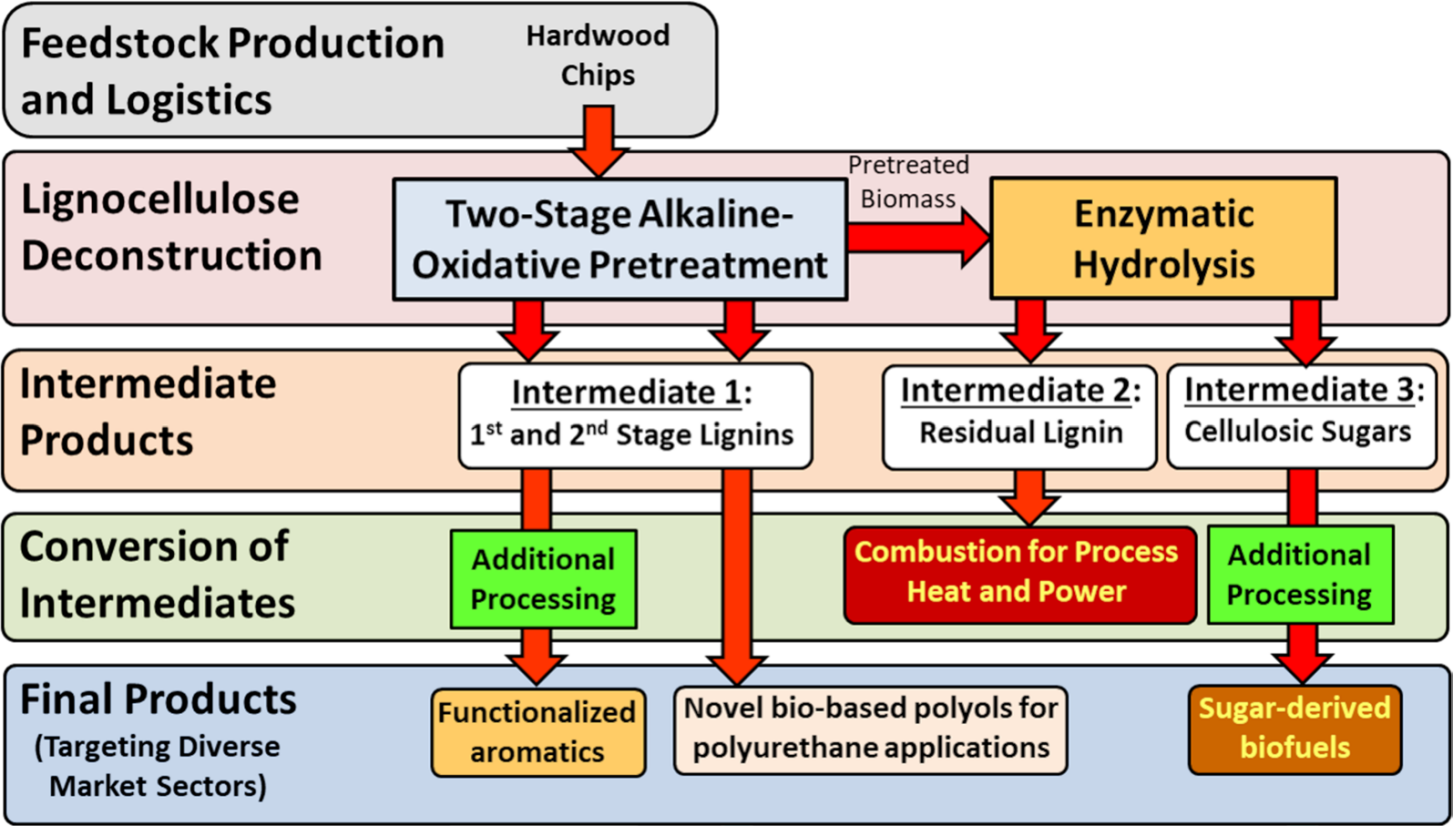
Overall biomass conversion pathway for generating lignin co-products and sugar-derived hydrocarbon biofuels

The overall processing strategy for biomass conversion to fuels and chemicals assessed in this work is presented in Fig. 2. The two-stage alkaline pre-extraction followed by alkaline-oxidative pretreatment method is used to fractionate lignocellulose biomass into various lignin and sugar streams for downstream conversion. As shown in Fig. 2, this approach provides the flexibility to accommodate shifting market conditions. It does this by yielding several lignin products that can target multiple markets, altering the properties of the lignin, and varying the partitioning of lignin into the three intermediate product pools, or target molecules.

One key set of target molecules includes phenolic acid and aldehyde monomers (vanillin, vanillic acid, syringaldehyde, syringic acid, and others) that can be directed towards high-value, low-volume markets (e.g., flavor and fragrance compounds). Specifically, the flavor and fragrance industry has a total global market size of $28 billion with strong continued growth forecasted in developing countries that tracks GDP [35]. As one example, synthetic vanillin is produced at the scale 37,000 tonnes/year primarily from petroleum-derived aromatics with a market value on the order $10/kg [36]. A small fraction of this market is bio-based vanillin derived from the alkaline oxidation of wood-derived spent sulfite black liquor and from the biological conversion of plant-derived ferulic acid [37]. This market offers a potential high-value niche for lignin-derived bio-based aromatics and a single biorefinery processing 2000 tonne/day of woody biomass would only displace 0.2% of this market. Another potential market for lignin-derived monomers includes medium-value, high-volume functionalized aromatic chemical markets [38–40], with applications that could include bio-based polymers such as a replacement for Bisphenol A in thermoset resins [41] and as novel bio-based monomer in polyesters [42]. A second class of target molecules is highly functionalized oligomeric lignins that have potential in resin formulations for bio-based polyurethane coatings. Well-established challenges for utilizing polymeric lignins as a feedstock for polyurethane resin production include its dark color, low solubility in reaction solvents, low reactivity, high polydispersity, and brittleness [9]. If these challenges can be overcome, process-derived lignins represent an opportunity as a renewable source of polyols in the production of polyurethane resin for coating applications. This unique and innovative approach for lignin depolymerization to yield aromatic monomers will yield a subset of lignins that are well-suited for application as polyols in polyurethane resin formulations with properties that include low molecular weights, low polydispersities, low glass transition temperatures, and high reactivities.

Unlike hydrogenation/reductive approaches to lignin depolymerization or conversion whereby alcohols, aldehydes, carboxylates, and aromatics are reduced and deoxygenated, oxidations can preserve and generate oxygen-containing functional groups (i.e., vanillin, vanillic acid, syringaldehyde, syringic acid, acetosyringone, acetovanillone). While the oxygen content of biomass-derived compounds is a negative for fuels applications, oxygen-containing groups are useful for providing chemical functionality and reactivity for use as platform chemicals or as reactive aromatic polymers that can be incorporated into polyurethane resin formulations to increase their bio-based content.

### Capital and operating costs

Table 4 shows the material balance for the eight pretreatment conditions assessed in this study based on our prior experimental study [23]. As shown, varying the pretreatment conditions impacted both the monomeric sugar (glucose and xylose) yields and the extent of delignification, thereby affecting the yields of biofuels and lignin-based products (polyols and aromatic monomers). With increasing pretreatment severity (i.e., temperature and oxidant loading), the yields of products (monomeric sugars and lignin) were increased (Additional file 1: Table S3). However, increasing the pretreatment severity also resulted in increased capital and operating costs. Therefore, an optimum balance between the process costs and the product yields needed to be identified with the technoeconomic model.

**Table 4:** Material balance of the studied conditions (feedstock: 2000 dry metric tonne/day)

Experimental reaction conditions^a^	Glucose	Xylose	Product generation (metric tonne/day)	
			Total solubilized lignin	Total precipitated lignin
120 °C—Cu-AHP 8% H_2_O_2_	822.1	320.1	225.1	135.2
120 °C—Cu(bpy) + O_2_	808.0	309.9	222.4	133.8
120 °C—Cu-AHP 8% H_2_O_2_ + O_2_	984.8	359.1	333.5	160.8
120 °C—Cu-AHP 6% H_2_O_2_ + O_2_	975.6	359.1	323.9	160.4
120 °C—Cu-AHP 4% H_2_O_2_ + O_2_	959.8	359.1	307.4	158.2
120 °C—Cu-AHP 2% H_2_O_2_ + O_2_	946.9	359.1	297.8	156.1
90 °C—Cu-AHP 8% H_2_O_2_	653.6	256.0	167.5	85.9
90 °C—Cu-AHP 4% H_2_O_2_ + O_2_	778.0	289.4	216.9	98.0

^a^120 and 90 °C: alkaline pre-extraction step conducted at 120 and 90 °C, respectively. Cu-AHP H_2_O_2_: Cu-AHP pretreatment performed at 80 °C; Cu(bpy) + O_2_: Cu(bpy)-catalyzed alkaline-oxidative pretreatment with 50 psig O_2_ as the only oxidant; Cu-AHP H_2_O_2_ + O_2_: O_2_-enhanced Cu-AHP pretreatment (50 psig O_2_). Values are expressed as averages of triplicate experiments

**Fig. 3:**
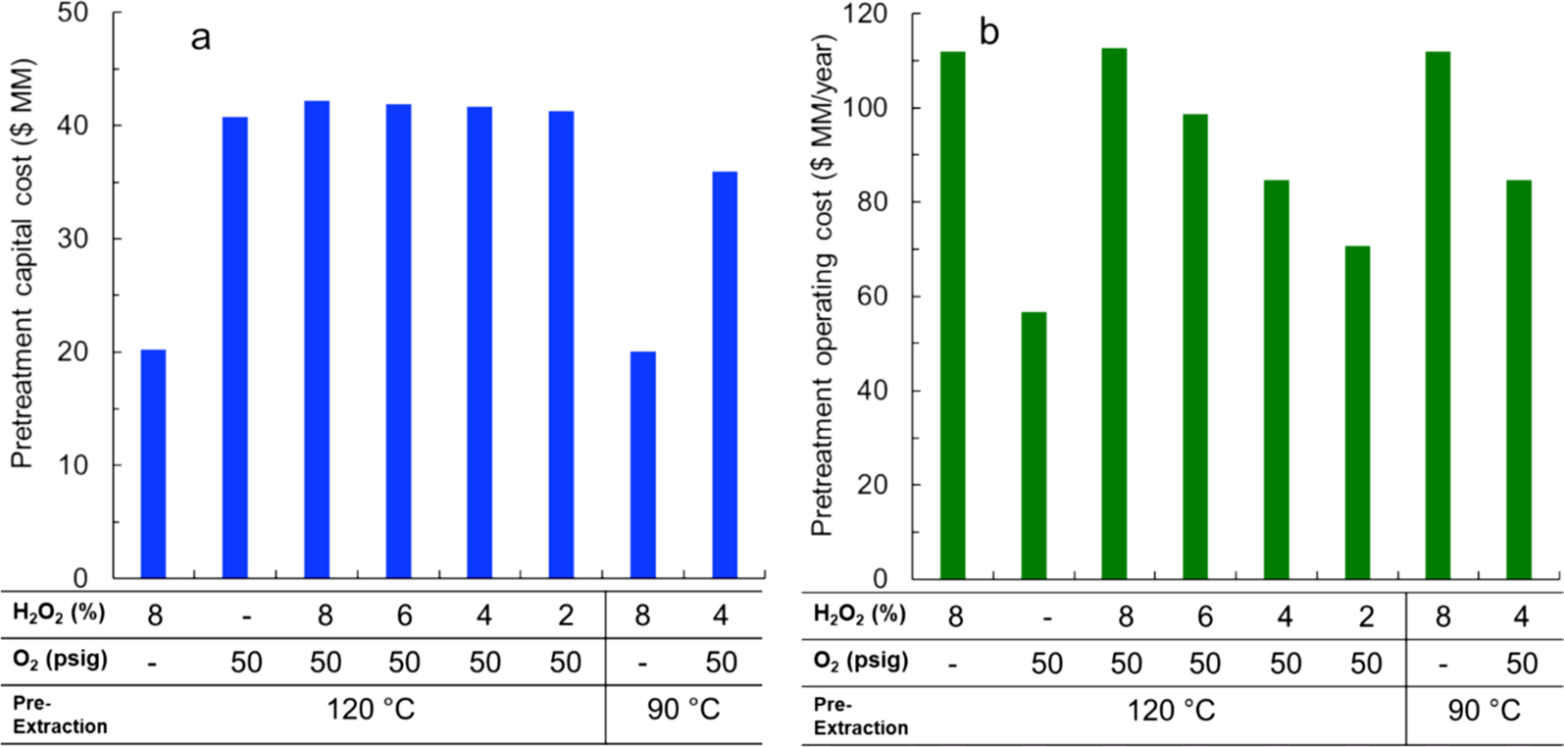
The pretreatment **a** capital cost and **b** operating cost of the poplar biorefinery

Figure 3a shows the total capital costs for all eight pretreatment conditions modeled in this study. The use of both H_2_O_2_ and O_2_ as co-oxidants during the alkaline-oxidative pretreatment stage increased the capital cost compared to the alkaline-oxidative pretreatment with H_2_O_2_ only. Moreover, the case with alkaline pre-extraction performed at 90 °C and alkaline-oxidative pretreatment performed with only 8% H_2_O_2_ had the lowest total capital cost ($20.1 million), while the case with alkaline pre-extraction performed at 120 °C and the alkaline-oxidative pretreatment with 8% H_2_O_2_ and 50 psig O_2_ had the highest total capital cost ($42.2 million). This could be attributed to the higher cost reactor; the addition of 50 psig O_2_ requires a much thicker vessel than the case without using O_2_.

Figure 3b displays the operating costs for the eight pretreatment conditions. As shown, under the same alkaline pre-extraction temperature (120 °C), using O_2_ in addition to H_2_O_2_ during the alkaline-oxidative pretreatment stage only slightly increases operating costs. O_2_ was assumed to be recovered from air on site, during which only the electricity was used as a contributor to the operating cost. In contrast, reducing H_2_O_2_ utilization from 8 to 2% reduced operating costs by $42 million/year due to the relatively high cost of purchasing H_2_O_2_ ($1/kg); this could lead to a considerable decrease in MFSP. To probe further the operating cost, the individual contributors to the operating cost were also investigated (Additional file 1: Table S4). Moreover, when using the solubilized lignin for high-value products instead of burning for energy, the required electricity increased for the cases that solubilized more lignin during the pretreatment process; this also increased the operating cost.

### Minimum fuel selling price (MFSP)

**Fig. 4:**
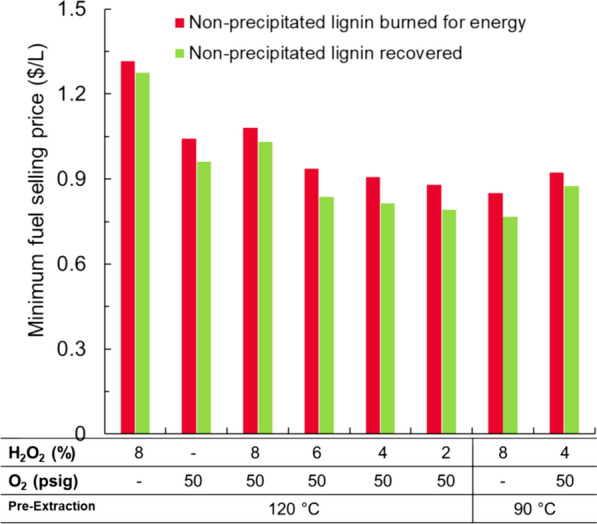
Minimum fuel selling price (MFSP) in $/L for various Cu-AHP pretreatment conditions. MFSP is shown assuming non-precipitated soluble lignin in the extract of the second pretreatment stage is either burned for energy (red bars) or recovered for high-value lignin products (green bars)

Figure 4 shows the estimated MFSP ($/L) for the eight pretreatment conditions considered in this study. Two scenarios are presented for the MFSP. In the first scenario, the soluble lignin that is not precipitated is burned for energy, while in the second scenario, the soluble lignin in the Cu-AHP extract that is not precipitated is assumed to be recoverable and sold at the same price as the precipitated lignin ($0.80/kg). The cost of pretreatment chemicals had a large influence on the MFSP, accounting for 40% of the total operating costs for the base case of a 120 °C alkaline pre-extraction followed by an alkaline-oxidative Cu-AHP pretreatment with 8% H_2_O_2_ (120 °C—Cu-AHP 8% H_2_O_2_). If we assumed that the acid-soluble lignin was not recoverable, the MFSP using H_2_O_2_ as the only oxidant [(120 °C—Cu-AHP 8% H_2_O_2_) and (90 °C—Cu-AHP 8% H_2_O_2_)] was between $1.32/L and $1.08/L depending on the temperature of the alkaline pre-extraction stage. Conversely, when O_2_ was used as a co-oxidant and the H_2_O_2_ loading was reduced from 8 to 2%, the MFSP decreased to between $0.94/L and $0.85/L. This is because this sizable reduction in pretreatment chemical usage did not result in a corresponding large reduction in sugar yields (Additional file 1: Table S2; [23]). Eliminating the H_2_O_2_ entirely led to slight increase in MFSP due to an appreciable reduction in both the sugar and lignin yields (Additional file 1: Table S2; [23]). Importantly, if the acid-soluble lignin can be recovered for value-added products, then the MFSP can be reduced by an additional $0.10/L (down to $0.77/L) if O_2_ is employed as a co-oxidant during the Cu-AHP process (120 °C—Cu-AHP 2% H_2_O_2_ + O_2_). The use of O_2_ as a co-oxidant increased the amount of lignin solubilized during pretreatment, but a larger proportion of this lignin was acid soluble. Thus, the difference in MFSP between the two assumptions (all solubilized lignin is recoverable versus only precipitated lignin) was greater when O_2_ was employed as a co-oxidant.

The TEA indicates that the overall MFSP can be reduced by nearly 40% by using O_2_ as a co-oxidant in the Cu-AHP process relative to the Cu-AHP pretreatment using H_2_O_2_ only. This is due both to a decrease in pretreatment operating cost (due to a reduction in H_2_O_2_ loading) and to an increase in both glucose and lignin yield. The primary tradeoff for oxygen utilization is a modest increase in electricity usage to generate the oxygen as well as an increase in capital costs (the oxygen production unit is assumed to cost $9.7 million, while the cost of increasing the pressure rating of the pretreatment vessel is $12.4 million). Despite these costs, the added capital cost only increased ~ 6% (Fig. 2) and therefore did not greatly impact the MFSP. From the results in Fig. 3, pretreatment conditions of alkaline pre-extraction (120 °C) and alkaline-oxidative pretreatment (2% H_2_O_2_ and O_2_) were selected as the base case for further analysis. Moreover, a detailed list of contributors to the MFSP of the selected base case (2% H_2_O_2_ and O_2_) was also provided (Additional file 1: Table S4).

### Effect of lignin valorization on MFSP

**Fig. 5:**
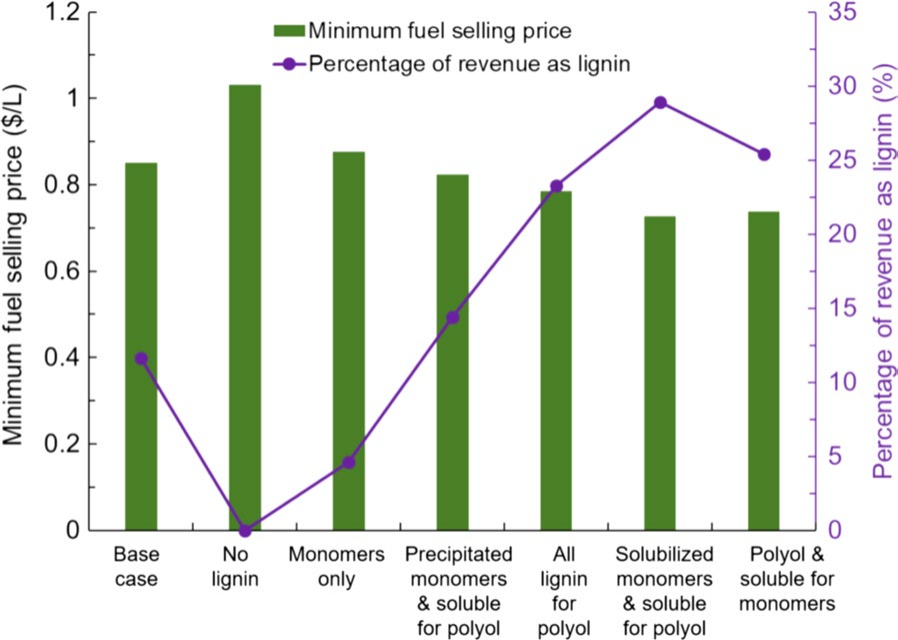
Impact of lignin recovery on minimum fuel selling price (MFSP) in $/L. The scenarios include (1) base case—precipitated lignin sold as a polyol replacement; (2) no lignin—no lignin recovered as value-added material; (3) monomers only—16% of precipitated lignin sold as high-value monomers with the remainder only for burning; (4) precipitated monomers and soluble for polyol—16% of precipitated lignin sold as high-value monomers with the remainder as a polyol replacement; (5) all lignin for polyol—all solubilized lignin sold as a polyol replacement; (6) solubilized monomers and soluble for polyol—16% of all solubilized lignin sold as high-value monomers with the remainder as a polyol replacement; (7) polyol and soluble for monomers—all precipitated lignin sold as a polyol replacement, while 48% of non-precipitated lignin sold as high-value monomers. Note that in all cases, any lignin not recovered as either polyol replacement or high-value monomers is burned in the boiler for heat and/or power

The above analysis assumed only lignin that was solubilized and recoverable by acid precipitation could be utilized as a polyol substitute. Multiple other scenarios were also considered: (1) no lignin was recovered for additional value as a worst-case scenario; (2) 16% of the recovered lignin (based on results obtained from lignin depolymerization following the method of sequential Bobbitt’s salt oxidation followed by formic acid-catalyzed depolymerization process [23]) could be sold as monomers, increasing its value to $2.00/kg, while the remainder of the precipitated lignin was burned for fuel; (3) the same 16% of recovered lignin is sold as monomers, but the remaining recovered lignin was sold as a polyol substitute; (4) the solubilized but not precipitated lignin could also be recovered and sold as a polyol substitute ($0.80/kg); (5) 16% of all solubilized lignin (including the non-precipitated portion) was sold as monomers (with the remainder as a polyol substitute), and (6) the precipitated lignin was sold as a polyol substitute, while 48% of the non-precipitated lignin was sold as monomers (Fig. 5).

Importantly, if the acid-soluble lignin can be recovered for value-added products, then the MFSP can be reduced by an additional $0.07/L (down to $0.78/L) if O_2_ is employed as a co-oxidant during the Cu-AHP process (120 °C—Cu-AHP 2% H_2_O_2_ + O_2_). As noted above, the use of O_2_ as a co-oxidant increased the amount of lignin solubilized during pretreatment, but a larger proportion of this lignin was acid soluble. Thus, a strategy to recover this soluble lignin will be important to further optimize this process due to the presence of oxygen. Likewise, if the value of the lignin can be increased by conversion to aromatic monomers, the MFSP can be reduced further to $0.73/L. This is due solely to increased value of lignin, as it increases from 12 to 26% of the total revenue of the biorefinery. An intermediate approach, in which 48% of the soluble lignin can be recovered and sold as high-value monomers, also significantly reduces the cost to $0.74/L. If lignin is not recovered as a co-product, the MFSP is $1.03/L, indicating the importance of lignin recovery during Cu-AHP pretreatment. In the case that the precipitated lignin is converted to monomers at a 16% yield but the remaining precipitated lignin can only be burned as fuel, the MFSP is only $0.88/L, less than the base case in which the precipitated lignin is used as a polyol substitute. Thus, while developing this technology, it is imperative that either yields for monomers increases or the process allows for the remaining lignin to be used as a polyol substitute.

Significant advances have been made to reduce the input costs of copper-catalyzed alkaline hydrogen peroxide pretreatment while simultaneously maintaining high sugar yields (95% glucose and ~ 100% xylose of initial sugar composition) [23, 43, 44]. Despite this, the operating costs for pretreatment were still high at approximately $71 million/year for a 2000 dry tonne/day facility (Fig. 4) or $97/tonne biomass, resulting in a $1.03/L MFSP if no lignin was recovered as a value-added product. This decreased to $0.85/L if precipitated lignin was recovered as a polyol substitute and $0.78/L if all soluble lignin could be recovered as a polyol substitute. While the technology to produce polyurethane products from lignin is relatively well understood, the possibility of producing monomers can reduce the selling price further down to $0.73/L. While challenges currently remain to commercializing this technology, it demonstrates that further selling price reductions are possible as improvements in lignin valorization continue. Thus, the combination of reduced pretreatment inputs while maintaining high sugar and lignin solubilization and improved usage of recovered lignin is instrumental in obtaining economically competitive biofuels.

### Sensitivity analysis

**Fig. 6:**
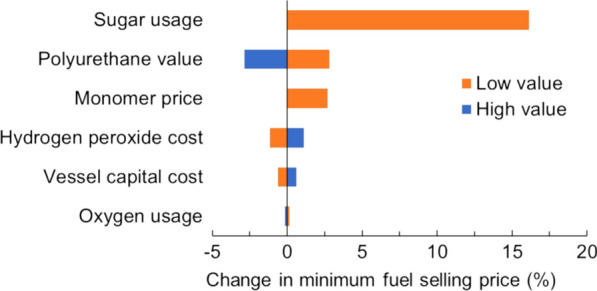
Sensitivity analysis results using scenarios for “low” and “high” outlined in Table 3

Understanding the impact of key parameters on the MFSP is of great importance to developing this technology further. Sensitivity of the MFSP with the sequential two-stage alkaline pre-extraction and alkaline-oxidative pretreatment of hybrid poplar (the selected base case) is summarized in Fig. 6, in which the capital and operating costs were also included. Yield of both sugar and lignin had the highest impact on the final biofuel selling price, indicating the importance of recovering all of the solubilized material. Likewise, the value of the lignin, used either in polyurethane applications or as lignin monomers, also resulted in large changes in the biofuel selling price. This indicates that revenue, rather than the individual costs of the refinery, drives the economics of the process. Each of the cost drivers selected, namely hydrogen peroxide cost, pretreatment capital cost, oxidation pressure, and total oxygen usage had relatively minimal impact on the final selling price of the fuel. This analysis provides evidence that, if the high yields and potentially high value for lignin can be maintained as the process is scaled to more industrially relevant conditions, the potential for economic value will remain even if costs are greater than initially anticipated.

The results further indicate that it is of great importance to include the lignin properties and valorization strategies when establishing TEA models for biorefinery. This is because the lignin value has a significant impact on the MFSP (based on the scenarios we studied). To include the lignin value in the model, there are several methods to be considered: (1) purifying lignin for the various applications in order to design downstream separations; (2) recycling or treating any waste streams generated from these separations, and (3) drying and packaging of the final lignin product. Synergies may be found if the final product (such as polyurethane) is produced at the same location. Further laboratory optimization of lignin separations and purification would also be required.

### Conclusions

Cu-AHP is promising technology to improve the production of biofuels from lignocellulose, and this economic assessment illustrates the importance of considering high-value co-products when assessing these technologies. The pretreatment process described herein demonstrated high sugar and lignin yields while reducing the raw material input in the pretreatment, thereby yielding biofuel at a cost as low as $0.85/L. In addition to the high yields, diversification of the lignin products into higher value products has potential to reduce the fuel costs even further to $0.73/L, compared to a value of $1.03/L if the lignin is only used as a fuel source. Given the promising results that both high-value lignin and high sugar yields can be obtained while significantly reducing the pretreatment costs, further research is thus warranted on improving and integrating the pretreatment and lignin valorization technologies, moving both of them to a more commercially ready state. Modeling this integration as the technology continues to progress will also be instrumental in optimizing the conditions and ensuring the process is economically viable.

## Supplementary Information

**Additional file 1: Table S1.** Chemical composition of poplar after alkaline pre-extraction. **Table S2.** Chemical composition of poplar following the two-stage alkaline-oxidative pretreatment process performed under various conditions. **Table S3.** Yields of glucose and xylose following enzymatic hydrolysis of the two-stage pretreated poplar biomass. **Table S4.** Operating cost summary for 120 °C alkaline pre-extraction, 2% H_2_O_2_ with 50 psig O_2_ for the second-stage alkaline-oxidative pretreatment (120 °C—Cu-AHP 2% H_2_O_2_ + O_2_).

## Data Availability

The datasets used and/or analyzed during the current study are available from the corresponding author on reasonable request.
