# Supplementary material for: Technoeconomic evaluation of recent process improvements in production of sugar and high-value lignin co-products via two-stage Cu-catalyzed alkaline-oxidative pretreatment
Source: Biotechnol Biofuels Bioprod. 2022 May 4;15:45. doi: 10.1186/s13068-022-02139-5 (PMC9069716; doi:10.1186/s13068-022-02139-5)
Supplement: Supplementary file 1 — Additional file 1: Table S1. Chemical composition of poplar after alkaline pre-extraction. Table S2. Chemical composition of poplar following the two-stage alkaline-oxidative pretreatment process performed under various conditions. Table S3. Yields of glucose and xylose following enzymatic hydrolysis of the two-stage pretreated poplar biomass. Table S4. Operating cost summary for 120 °C alkaline pre-extraction, 2% H2O2 with 50 psig O2 for the second-stage alkaline-oxidative pretreatment (120 °C—Cu-AHP 2% H2O2 + O2). [file 13068_2022_2139_MOESM1_ESM.docx]

**Supporting Information**

**Technoeconomic Evaluation of Recent Process Improvements in Production of Sugar and High-Value Lignin Co-products via Two-Stage Cu-Catalyzed Alkaline-Oxidative Pretreatment**

Zhaoyang Yuan^1, #^, Bryan D. Bals^2, #,^*, Eric L. Hegg^1,^*, David B. Hodge^3,4,^*

1. Department of Biochemistry & Molecular Biology, Michigan State University, 603 Wilson Road, East Lansing, Michigan 48824, United States

2. Michigan Biotechnology Institute, 3815 Technology Boulevard, Lansing, Michigan 48910, United States

3. Department of Chemical & Biological Engineering, Montana State University, Bozeman, Montana 59717, United States

4. Division of Sustainable Process Engineering, Luleå University of Technology, Luleå, Sweden

#Equal contribution

*Corresponding author:

[bryanbals@gmail.com](mailto:bryanbals@gmail.com) (Bryan Bals)

[erichegg@msu.edu](mailto:erichegg@msu.edu) (Eric Hegg)

[david.hodge3@montana.edu](mailto:david.hodge3@montana.edu) (David Hodge)

The Supporting Information consists of 5 pages, including 4 tables:

Table S1······································································································S2

Table S2······································································································S3

Table S3······································································································S4

Table S4······························ ·······································································S5

**Supplementary Tables**

**Table S1** Chemical composition of poplar after alkaline pre-extraction

| Substrate | Original | 90 °C | 120 °C |
| --- | --- | --- | --- |
| Solid remaining (%)^a^ | N/A | 87.1 ± 0.1 | 78.5 ± 0.1 |
| Glucan (%) | 45.5 | 50.1 (43.6)^b^ ± 0.8 | 54.7 (42.9)^b^ ± 1.2 |
| Xylan (%) | 15.8 | 16.4 (14.3)^b^ ± 0.2 | 15.5 (12.2)^b^ ± 0.2 |
| Klason lignin (%) | 22.3 | 23.3 (20.3)^b^ ± 0.3 | 21.1 (16.6)^b^ ± 0.4 |
| Ash (%) | 0.85 | 0.87 (0.76)^b^ ± 0.1 | 0.86 (0.68)^b^ ± 0.1 |

N/A: Not applicable. Values are expressed as an average ± standard deviation of triplicate experiments.

^a^ Weight percentage (oven-dried weight) of the recovered biomass after alkaline pre-extraction relative to original biomass.

^b^ Chemical composition of alkaline pre-extracted biomass relative to original biomass.

**Table S2** Chemical composition of poplar following the two-stage alkaline-oxidative pretreatment process performed under various conditions

| Experiment^a^ | Solid Remaining (%)^b^ | Glucan (%)^c^ | Xylan (%)^c^ | Acid-Insoluble Lignin (%)^c^ |
| --- | --- | --- | --- | --- |
| Untreated | N/A | 45.5 | 15.8 | 22.3 |
| 120 °C – Cu-AHP 8% H_2_O_2_ | 68.6 ± 0.7 | 58.1 ± 0.6 | 14.2 ± 0.5 | 16.1 ± 0.5 |
| 120 °C – Cu(bpy) + O_2_ | 69.6 ± 0.7 | 56.8 ± 0.8 | 13.6 ± 0.3 | 16.3 ± 0.3 |
| 120 °C – Cu-AHP 8% H_2_O_2_ + O_2_ | 52.2 ± 0.6 | 74.7 ± 1.1 | 12.9 ± 0.6 | 8.2 ± 0.5 |
| 120 °C – Cu-AHP 6% H_2_O_2_ + O_2_ | 52.6 ± 0.6 | 74.4 ± 0.9 | 13.1 ± 0.5 | 8.9 ± 0.4 |
| 120 °C – Cu-AHP 4% H_2_O_2_ +O_2_ | 53.2 ± 0.4 | 73.7 ± 1.2 | 13.0 ± 0.6 | 10.1 ± 0.5 |
| 120 °C – Cu-AHP 2% H_2_O_2_ + O_2_ | 54.1 ± 0.8 | 73.3 ± 1.3 | 12.9 ± 0.5 | 10.8 ± 0.4 |
| 90 °C – Cu-AHP 8% H_2_O_2_ | 76.3 ± 0.6 | 54.6 ± 0.9 | 15.1 ± 0.6 | 20.3 ± 0.6 |
| 90 °C – Cu-AHP 4% H_2_O_2_ + O_2_ | 71.4 ± 0.8 | 57.9 ± 1.1 | 13.1 ± 0.3 | 16.7 ± 0.8 |

N/A: Not applicable.

^a^ 120 °C and 90 °C: alkaline pre-extraction step conducted at 120 °C and 90 °C, respectively. Cu-AHP H_2_O_2_: Cu-AHP pretreatment performed at 80 °C; Cu(bpy) + O_2_: Cu(bpy)-catalyzed alkaline-oxidative pretreatment with 50 psig O_2_ as the only oxidant; Cu-AHP H_2_O_2_ + O_2_: O_2_-enhanced Cu-AHP pretreatment using 50 psig O_2_. Values are expressed as average ± standard deviation of triplicate experiments.

^b^ Weight percentage (oven-dried weight) of the recovered biomass after two-stage Cu-AHP pretreatment relative to original biomass.

^c^ Weight percentage based on oven-dried weight of two-stage pretreated biomass.

**Table S3** Yields of glucose and xylose following enzymatic hydrolysis of the two-stage pretreated poplar biomass

| Experiment^a^ | Glucose (%)^b^ | Xylose (%)^b^ |
| --- | --- | --- |
| 120 °C – Cu-AHP 8% H_2_O_2_ | 68.9 ± 0.8 | 50.8 ± 0.9 |
| 120 °C – Cu(bpy) + O_2_ | 66.8 ± 0.9 | 46.2 ± 0.8 |
| 120 °C – Cu-AHP 8% H_2_O_2_ + O_2_ | 83.1 ± 1.1 | 48.3 ± 1.1 |
| 120 °C – Cu-AHP 6% H_2_O_2_ + O_2_ | 82.5 ± 1.1 | 46.5 ± 1.2 |
| 120 °C – Cu-AHP 4% H_2_O_2_ +O_2_ | 81.1 ± 1.3 | 45.8 ± 0.9 |
| 120 °C – Cu-AHP 2% H_2_O_2_ + O_2_ | 80.8 ± 1.1 | 46.2 ± 0.8 |
| 90 °C – Cu-AHP 8% H_2_O_2_ | 56.2 ± 0.7 | 44.2 ± 1.0 |
| 90 °C – Cu-AHP 4% H_2_O_2_ + O_2_ | 67.8 ± 1.4 | 39.8 ± 0.7 |

^a^ 120 °C and 90 °C: alkaline pre-extraction step conducted at 120 °C and 90 °C, respectively. Cu-AHP H_2_O_2_: Cu-AHP pretreatment performed at 80 °C; Cu(bpy) + O_2_: Cu(bpy)-catalyzed alkaline-oxidative pretreatment with 50 psig O_2_ as the only oxidant; Cu-AHP H_2_O_2_ + O_2_: O_2_-enhanced Cu-AHP pretreatment using 50 psig of O_2_.

^b^ The sugar yields were calculated based original sugar composition in untreated biomass.

Values are expressed as average ± standard deviation of triplicate experiments.

**Table S4** Operating cost summary for 120 °C alkaline pre-extraction, 2% H_2_O_2_ with 50 psig O_2_ for the second-stage alkaline-oxidative pretreatment (120 °C – Cu-AHP 2% H_2_O_2_ + O_2_)

| Cost Item | Cost ($/liter biofuel) | Assumption |
| --- | --- | --- |
| Labor | $ 0.008 | 38 total employees |
| Maintenance | $ 0.016 | 3% of IBSL |
| Insurance | $ 0.011 | 0.7% of fixed capital investment |
| Hybrid poplar | $ 0.10 | $55.1/metric tonne |
| NaOH | $ 0.04 | $149/metric tonne |
| CuSO_4_ | $ 0.063 | $1.50/ kg |
| H_2_O_2_ | $ 0.037 | $1.00/kg |
| 2,2′-bipyridine | $ 0.085 | $30/kg |
| H_2_SO_4_ | $ 0.04 | $88/metric tonne |
| Glycoside hydrolase enzymes | $ 0.063 | $5.00/kg |
| H_2_ | $ 0.21 | $1.50/kg |
| Catalyst | $ 0.029 | From Davis et al. 2015^a^ |
| Catalyst regeneration chemicals | $ 0.013 | From Davis et al. 2015^a^ |
| Other chemicals | $ 0.003 | From Davis et al. 2015^a^ |
| Makeup water | $ 0.003 | From Davis et al. 2015^a^ |
| Ash disposal | $ 0.003 | $39/metric tonne |
| Electricity | $ 0.034 | 6.5 cents/kWh |
| Lignin co-product | $ (0.11) | $0.80/kg, soluble lignin not included |
| Total operating cost | $ 0.65 |  |
| Income, taxes, depreciation | $ 0.20 |  |
| **Minimum fuel selling price (MFSP)** | **$ 0.85** |  |
